# Isolation and identification of lactic acid bacteria from ginseng sprouts and research on their probiotic, anti-inflammatory, and anti-cancer activity

**DOI:** 10.3389/fnut.2025.1718524

**Published:** 2026-01-05

**Authors:** JeongOk Lee, Selin Jung, Ji-Eun Lee, Muhammad Hamayun, Su Hwan Park, Young-Jin Choi, Eun-Kyung Kim, Jong-Ho Lee, Ho-Youn Kim, Bokyung Lee

**Affiliations:** 1 Department of Food Science and Nutrition, Dong-A University, Busan, Republic of Korea; 2 Department of Health Sciences, The Graduate School of Dong-A University, Busan, Republic of Korea; 3 Department of Botany, Abdul Wali Khan University, Mardan, Pakistan; 4 Nutritional Education Major, Graduate School of Education, Dong-A University, Busan, Republic of Korea; 5 Smart Farm Research Center, Korea Institute of Science and Technology (KIST), Gangneung, Republic of Korea; 6 Natural Product Applied Science, KIST School, University of Science and Technology (UST), Gangneung, Republic of Korea

**Keywords:** probiotics, *Lacticaseibacillus*, *Leuconostoc*, plant, anti-inflammation, TNF-α expression, antioxidants, anti-cancer activity

## Abstract

**Introduction:**

Ginseng sprouts are recognized as a potentially valuable food source as they provide an abundance of bioactive compounds with antioxidants and anti-cancer properties. This study investigates the probiotic properties of lactic acid bacteria (LAB) from ginseng sprouts, addressing gaps in plant-based probiotic discovery.

**Methods:**

A total of 17 putative LAB strains were obtained from 688 bacterial isolates from ginseng sprouts. Molecular identification via 16S rRNA sequencing classified and selected five isolates as LAB taxa. Their probiotic potential was evaluated through *in vitro* assays assessing gastrointestinal stress tolerance (pH 3.0 and 0.3% bile salt), antibacterial activity, safety, and antioxidant activity (DPPH scavenging), anti-inflammatory (TNF-α secretion), and cancer cell cytotoxicity (WST-8 assay and apoptosis induction).

**Results:**

After 16S rRNA sequencing, five candidate probiotic strains from ginseng sprouts were identified, including three strains of *Lacticaseibacillus rhamnosus* (B7112, B3402, and B3421), one strain of *Leuconostoc lactis* (B34171), and one strain of *Leuconostoc mesenteroides* (B22051). *In vitro* assays evaluating their probiotic potential revealed that all five strains exhibited robust tolerance to acidic pH and the presence of bile salts, notable antibacterial activity, antibiotic susceptibility, and strong adhesion to intestinal epithelial cells. Notably, these LAB isolates demonstrated DPPH radical scavenging activity comparable to that of 25 μM L-ascorbic acid, indicating significant antioxidant capacity. Furthermore, quantitative RT-PCR analysis showed that these LAB strains significantly downregulated TNF-α mRNA expression, reflecting substantial anti-inflammatory effects. In addition, protein extracts derived from LAB strains effectively inhibited cancer cell proliferation *in vitro*.

**Conclusion:**

These findings highlight the therapeutic promise of plant-derived probiotic bacterial strains for potential applications in human health, particularly in inflammation and cancer prevention. Moreover, the successful isolation of probiotic LAB from ginseng sprouts underscores the potential of ginseng as a valuable source of health-promoting microbiota.

## 1 Introduction

Probiotics are defined as live microorganisms that, when administered in adequate amounts, confer a health benefit on the host (1). Previous studies have reported that probiotic microorganisms may originate from conventional sources (healthy human digestive tract) or unconventional sources, such as the digestive tract of animals, breast milk, food (fermented or unfermented), air, or soil (2). The most common species of probiotics are lactic acid bacteria (LAB) such as *Lactobacillus* species, *Lacticaseibacillus* species, and *Bifidobacterium* species (3, 4). Additionally, species from the genera *Leuconostoc, Streptococcus, Propionibacterium, Bacillus, Enterococcus, Saccharomyces*, and others are also recognized as probiotics (3, 4). Probiotic bacteria help balance the gut microbiota (5), reduce obesity, alleviate inflammatory bowel diseases, inhibit intestinal pathogens, modulate immune responses, and decrease lactose intolerance, among other benefits (6). Additionally, they function as antioxidants by chelating metal ions, producing metabolites such as lactate, downregulating reactive oxygen species (ROS)-producing enzymes, and enhancing antioxidant activities (5). These actions help improve the host’s defenses against oxidative stress and play a vital role in preventing various diseases, including digestive disorders and cancer (6). As a result, the probiotic market has seen significant growth over the years (7).

In recent years, the increase in the incidence of inflammatory diseases has raised concerns worldwide. The majority of inflammatory diseases result from chronic inflammation that disrupts metabolic functions and induces cellular stress (8). In recent years, chronic inflammation has emerged as a central pathological mechanism underlying numerous health conditions, such as atherosclerosis, metabolic disorders such as obesity and type 2 diabetes, respiratory diseases, neurodegeneration, autoimmune diseases, and various malignancies (9). Inflammation is characterized by the activation of immune and non-immune cells in response to damage to internal organs (e.g., infection, hormone imbalance, and organ dysfunction) or external stimuli (e.g., pathogenic microbes or foreign particle invasion) (10). Inflammatory signaling at the cellular level is recognized as cells stimulate the production of pro-inflammatory cytokines (11), mainly including tumor necrosis factor-α (TNF-α), interleukin (IL)-6, IL-1, and IL-18, followed by triggering upstream or downstream protein expression and causing damage to the body (12). Another inflammatory mediator, nitric oxide (NO), also interacts with immune cells to amplify the inflammatory response (13). It has been reported that the excessive release of pro-inflammatory cytokines can cause acute or chronic inflammatory diseases (14). Numerous studies have shown that probiotic LAB strains can modulate the host’s immune response by influencing the production of cytokines involved in regulating and activating immune cells. Therefore, more effective probiotics need to be explored from valuable food sources to provide more alternatives in preventing inflammation.

Plant-derived probiotics have recently become of interest for consumers with lactose intolerance and for vegans in the probiotic market (15). Ginseng sprouts have recently gained recognition as a potentially valuable food source due to their abundance of bioactive compounds with anti-inflammatory and anti-cancer activity, such as ginsenosides and amino acids (16–18). A recent study has reported the presence of LAB, such as *Lactiplantibacillus plantarum*, *Lactobacillus gasseri*, *Limosilactobacillus reuteri*, *Ligilactobacillus salivarius*, *Streptococcus thermophilus*, and *Lactococcus lactis*, in the phyllosphere of hydroponically grown ginseng (19). This confirms the potential of ginseng sprouts as a source of probiotics. Although previous studies have reported the probiotic potential of strains isolated from ginseng, including *L. rhamnosus* (20), *L. casei* KGC1201 (21), *Limosilactobacillus fermentum* KGC1601 (22), and *Limosilactobacillus reuteri* KGC1901 (23), only one study has demonstrated its anti-inflammatory potential (22), while the anti-cancer potential of ginseng-originated LAB has not yet been reported.

Ginseng sprouts have been developed as medicinal vegetables or foods due to their relatively short growth period in a soil-less cultivation system without pesticides (24). Moreover, they are mostly consumed raw. Screening for probiotics in raw edible plants not only expands the diversity of functional strains (25) but also leverages the synergistic health benefits of plants and probiotics (26).

Our current study aimed to identify and characterize LAB strains isolated from ginseng sprouts, focusing on their *in vitro* properties related to anti-inflammatory and anti-cancer activities. In contrast to conventional approaches, this study examined various LAB species isolated from ginseng sprouts grown in a smart-farming system, offering a sustainable and controlled source of novel probiotic candidates.

## 2 Materials and methods

### 2.1 Plant material and growth conditions

Seeds of the Seosan cultivar of Korean ginseng (V3) and landrace ginseng (V4) were procured from the Rural Development Administration (RDA) and grown in the Smart Farm at Korea Institute of Science and Technology (KIST), Gangneung, Republic of Korea. The ginseng sprouts were cultivated as reported previously (19). Briefly, the seeds were hydroponically grown under controlled conditions (light intensity: 65–70 μmol/m^2^, photoperiod: 16 h/8 h day/night, temperature: 24 °C/18 °C day/night). Polyurethane sponge media and RDA’s approved hydroponic nutrient solution (EC 2.5, pH 6.8) were used. After 4 weeks, the seedlings were transferred to different LED chambers for light treatment (blue, infrared, red, and white). We collected ginseng sprouts grown under four different LED treatments from the Smart Farm at KIST and stored them at 4 °C.

### 2.2 Isolation of LAB from ginseng sprouts

To isolate LAB, ginseng sprouts were weighed, and a 0.1% (w/v) peptone (BD Difco, Detroit, USA) solution was added according to the sample weight (19). Ten-fold serial dilutions were performed for both sonicated peel and blended pulp samples with 0.1% peptone solution. These dilutions were spread on de Man, Rogosa, and Sharpe (MRS; BD Difco, Detroit, USA, Cat. no #288130) agar plates and incubated aerobically at 37 °C for 48 h. After incubation, single colonies were picked from the plates and cultured on fresh MRS agar plates. For long-term storage, the LAB isolates were mixed with glycerol (final concentration of 25%) and stored at −80 °C. *Lacticaseibacillus rhamnosus* GG KCTC 5033 (LGG) and *Lactobacillus acidophilus* KCTC 3164 were purchased from the Korean Collection for Type Cultures (KCTC), Jeonju, Republic of Korea and used as controls. LAB strains were grown in MRS broth (final pH 6.5, BD Difco, Detroit, USA, Cat. no #288130) and incubated at 37 °C for 18 h.

### 2.3 Identification and phylogenetic analysis of selected LAB isolates

To classify the presumed LAB, the MRS-grown isolates were cultured on bromocresol purple (BCP) agar plates (MB Cell, Seoul, Republic of Korea) at 37 °C for 18 h. Catalase test and Gram staining were performed for BCP-positive strains, as previously described (27). In total, 17 isolates that were MRS (+), BCP (+), catalase (−), and Gram (+), were selected to be presumed LAB and identified via 16S rRNA sequencing analysis and subsequent phylogenetic analysis. The 16S rRNA gene sequences of the B7112, B34171, B3421, B3402, and B22051 strains were deposited in the NCBI GenBank database under the accession numbers PQ276990, PQ276991, PQ276994, PQ276993, and PQ276995, respectively. The evolutionary history of the five selected isolates identified as LAB was inferred using the Neighbor-Joining method (28). Evolutionary analyses were conducted using the Mega software version 11 (29).

### 2.4 Probiotic characterization of the LAB strains

#### 2.4.1 Resistance to gastrointestinal tract (GIT)-related stressors, including pH, ethanol, and H_2_O_2_

The effects of several GIT related stressors, including pH, ethanol, and H_2_O_2_, were examined using a 96-well plate reader (BioTek, Synergy H1, Vermont, USA). Briefly, the selected LAB (strains #1, #2, #3, #4, and #5) were incubated at different pH levels (5 and 6.5), ethanol content (8%), and H_2_O_2_ (1 mM) in fresh MRS broth at 37 °C. The optical density at 600 nm (OD_600_) was measured and recorded every hour for each cell culture, with LGG used as a control.

#### 2.4.2 Tolerance of acidic pH and bile salts

Tolerance to low pH and bile content was assessed as previously described by Delgado et al. (30), with minor modifications. MRS broth was prepared, and the pH was adjusted to 3.0 with 1 N HCl (Duksan, Seoul, Republic of Korea) prior to use in the experiments. Acid tolerance was evaluated in acidified MRS broth (final pH 3.0), while bile tolerance was determined in MRS broth containing 0.3% bile salts (Sigma-Aldrich, St. Louis, MO, USA). Briefly, overnight cultures were harvested by centrifugation, and cells were suspended in MRS broth to obtain an OD_600_ < 1.0. Cell suspensions were diluted 10-fold with each type of modified MRS medium. pH and bile tolerance were evaluated by measuring survival after incubation at 37 °C for 3 and 6 h, respectively. The tolerance of the isolated bacteria was determined by enumerating the viable cells on MRS agar plates. Each assay was conducted in triplicate, and LGG was used as a positive control.

#### 2.4.3 Analysis of LAB resistance to antibiotics

Antibiotic resistance of LAB strains was evaluated using the protocol of Georgieva et al. (31), with minor adjustments. Briefly, approximately 10^8^–10^9^ CFU/ml of overnight cultures were spread on the MRS agar plates. Six antibiotic strips (bioMérieux SA, Marcy-l’Étoile, France) for erythromycin (EM, 0.016–256 μg/ml), tetracycline (TC, 0.016–256 μg/ml), clindamycin (CM, 0.016–256 μg/ml), gentamicin (GM, 0.016–256 μg/ml), benzylpenicillin (BP, 0.002–32 μg/ml), and ciprofloxacin (CF, 0.002–32 μg/ml) were applied to the plates. After 48 h incubation at 37 °C, antibiotic susceptibility or resistance was evaluated based on the inhibitory zones surrounding the antibiotic strips on the agar plates.

#### 2.4.4 Hemolysis test of LAB strains

The hemolytic activity of isolates was determined by incubating them at 37 °C for 48 h using agar plates containing 5% (w/v) sheep blood (KisanBio, Seoul, Korea) (32). The hemolytic phenotype around the colonies was observed. After incubation, the agar plates were examined for signs of β-hemolysis (clear zone around colonies), α-hemolysis (green colored zones around the colonies), or γ-hemolysis (no clear zone). *S. aureus* KCTC 3881 was used as a positive control.

#### 2.4.5 Antibacterial activity of LAB strains

The antibacterial activity of the LAB strains was assessed following the method published by Yerlikaya et al. (33, 34), with minor modifications. The bacterial strains used for comparison (*Staphylococcus aureus* KCTC 3881 and *Escherichia coli* KCTC 2593) were purchased from the KCTC. Pathogens were grown in nutrient broth (NB; BD Difco, Detroit, USA, Cat. no #234000) at 37 °C for 18 h. LAB cultures were grown in fresh MRS broth at 37 °C for 18 h, centrifuged (LABOGENE 1580R, Gyrozen, Daejeon, Republic of Korea) at 8,000 rpm for 5 min at 4 °C, and the supernatants were obtained, which were filtered through a 0.45 μm syringe filter (PVDF, sterile, Φ30mm, Biofil, China, Cat. no #J1.F403.030N). The resulting filtrate was used as the cell-free supernatant (CFS). Next, 8-mm paper disks (Toyo Roshi Kaisha, Ltd., Tokyo, Japan) were submerged in the CFS and stored at 4 °C for 14 h. Indicator cultures (100 μl) were spread on NB agar plates, and the treated paper disks were placed on the surface. The plates were then incubated for 18 h at 37 °C to assess the inhibition zones. Fresh MRS served as a negative control, and LGG/MRS (pH 4.0) served as a positive control. The pH of the stationary-phase cultures of our strains was 4.0, and MRS (pH 4.0) was used as a control to exclude pH-related effects. Each test was performed in triplicate.

#### 2.4.6 Evaluation of adhesion-related traits

##### 2.4.6.1 Auto-aggregation assay

Auto-aggregation activity was measured using the method of Polak-Berecka et al. (35). Briefly, the bacterial cells cultured in MRS broth for 18 h were centrifuged at 10,000 rpm for 10 min at 4 °C. After three washes with 1 × phosphate-buffered saline (PBS, T&I Co., Ltd., Seoul, Republic of Korea), the pellet was suspended in PBS. The absorbance at a wavelength of 600 nm was adjusted to 0.5–0.6 to standardize the number of bacteria. Bacterial suspensions (4 ml) were thoroughly vortexed for 15 s and incubated at 37 °C for 5 h. The OD_600_ was measured at the start and end of incubation. Auto-aggregation (%) was calculated as:


$$\text{Auto}-\text{aggregation}\;(\%)=(1-\frac{\text{Absorbance}\;\mathrm{at}\;\text{final point}}{\text{Absorbance}\;\mathrm{at}\;\text{initial point}})\times 100$$

LGG and *L. acidophilus* KCTC 3164 were used as positive controls.

##### 2.4.6.2 Cell-surface hydrophobicity assessment

LAB strains were grown for 16 h at 37 °C in fresh MRS broth to assess their ability to adhere to ethyl acetate, following the method of Abbasiliasi et al. (36). Cells in overnight cultures were harvested via centrifugation at 10,000 rpm for 10 min at 4 °C. After washing the cell pellet three times in 1 × PBS, 3 ml of the cell suspension adjusted to an OD_600_ of 0.6–0.7 was prepared for further studies. Next, 1 ml of ethyl acetate (Samchun, Ansan, Korea) was added to 3 ml of the cell suspension. After 1 min of vortexing, the mixture was reacted at room temperature (22 °C) for 10 min to separate the aqueous phases. The absorbance of the aqueous phase at 600 nm was determined. Hydrophobicity (%) was calculated as follows:


$$\text{Hydrophobicity}\;(\%)=(1-\frac{\mathrm{At}}{A0})\times 100$$

*A0* and *At* represent the initial absorbance and the absorbance at 10 min, respectively. LGG and *L. acidophilus* KCTC 3164 were used as positive controls.

#### 2.4.7 DPPH radical scavenging (antioxidant assay)

DPPH radical scavenging activity was assessed following the method published by Chen et al. (37) with minor modifications. Briefly, after 16 h of incubation, the bacterial culture was centrifuged at 12,000 rpm for 10 min at 4 °C. The cell pellets were washed thrice with 1 × PBS and combined with 4.0 mM DPPH solution (Thermo Fisher Scientific, Waltham, MA, USA) in methanol (1:1). The mixture was incubated for 30 min at 37 °C in the dark. Centrifugation was carried out at 12,000 rpm for 5 min at 4 °C. The supernatant was loaded in a 96-well plate, and the absorbance at 517 nm was measured. A solution of L-ascorbic acid (Sigma-Aldrich, St. Louis, MO, USA) and the LGG strain was used as a positive control. The DPPH radical scavenging activity was calculated as follows:


DPPH radical scavenging activity(%)=(Ac−As)/Ac×100

where Ac and As represent the control and sample absorbance, respectively.

### 2.5 TNF-α assay on stimulated RAW 264.7 cells by selected LAB strains

#### 2.5.1 Preparation of protein extracts (PE) from LAB

PE sample preparation was started by inoculating a single colony in 50 ml of MRS broth. The culture was incubated at 37 °C for 18 h and then centrifuged (12,000 rpm, 4 °C, 10 min) to separate the pellet and the supernatant. The pellets were washed two times with sterile ice-cold 1 × PBS and suspended in 1 ml of 1 × PBS. The cell suspension was transferred to a bead tube (Lysing Matrix B, MP Biomedicals, CA, USA) and lysed with a Bead Beater (FastPrep-24, MP Biomedicals, CA, USA) homogenizer (speed 6.0 m/s) by alternating three cycles of 40 s of lysing and 1 min of refrigeration in an ice bath. After homogenization, centrifugation (13,000 rpm, 4 °C, 5 min) was performed to collect the PE, which comprises soluble proteins in the supernatant. The protein concentrations were determined using the BCA protein assay kit (Thermo Fisher Scientific, Waltham, MA, USA) according to the manufacturer’s instructions. The PE samples were stored at −80 °C until use.

#### 2.5.2 RNA extraction and RT-PCR to evaluate anti-inflammatory activity

RAW 264.7 cells (KCLB number: 40071) were obtained from the Korean Cell Line Bank (Seoul, Republic of Korea). They were cultured in DMEM (Welgene, Gyeongsan, Republic of Korea) containing 10% FBS (Welgene, Gyeongsan, Republic of Korea) and 1% penicillin/streptomycin (P/S, Gibco, Grand Island, NY, USA) in a humidified atmosphere of 5% CO_2_ at 37 °C. The cells were then seeded into 12-well plates at a density of 5 × 10^5^ cells/well and incubated in a humidified atmosphere of 5% CO_2_ at 37 °C for 24 h. After treatment with lipopolysaccharide (lipopolysaccharide (LPS, 100 ng/ml, Cat. no #L2630-10mg; Sigma Aldrich, St. Louis, MO, USA) and PE of LAB (25 and 50 μg/ml) for 24 h, the cells were harvested. The PE of LGG at the same concentration served as a positive control. RNA was extracted using TRIzol reagent (Invitrogen, Carlsbad, CA, USA). To guarantee total cell disruption, 1 ml of TRIzol reagent was added to each well of the culture dish, and the cells were lysed immediately. After transferring the lysate to a microcentrifuge tube, 200 μl of chloroform (Duksan Pure Chemicals, Ansan, Korea) was added in order to isolate the RNA. The RNA-containing aqueous phase was collected, centrifuged at 13,000 rpm and 4 °C for 20 min, and isopropanol (Daejung Chemical Co., Siheung, Republic of Korea) was used to precipitate the RNA. Next, 1 ml of 75% ethanol diluted with diethylpyrocarbonate (DEPC)-treated distiled water (Invitrogen, Carlsbad, CA, USA) was added and centrifuged at 13,000 rpm at 4 °C for 5 min. Afterward, the supernatant was removed, and the microtube was turned over and allowed to dry for 30 min. Subsequently, DEPC-treated water was used to dissolve the RNA. A NanoDrop spectrophotometer (Thermo Fisher Scientific, Wilmington, DE, USA) was used to measure the concentration and purity of the extracted RNA. RNA samples having A260/A280 ratios between 1.8 and 2.0 were regarded as pure. An AccuPower RT PreMix (Bioneer, Daejeon, Republic of Korea) was used to synthesize cDNA. TNF-α and GAPDH expression were determined via quantitative PCR using a MIC qPCR cycler (Bio Molecular Systems, Upper Coomera, QLD, Australia). Primers were designed for TNF-α (sense, AAG CCT GTA GCC CAC GTC GTA; anti-sense, GGC ACC ACT AGT TGG TTG TCT TTG) and GAPDH (sense, GCA CAG TCA AGG CCG AGA AT; anti-sense, GCC TTC TCC ATG GTG GTG AA). Using the Ct value given by the micPCR program, the △Ct value was calculated as the target gene Ct–GAPDH Ct value and expressed as 2^-△△Ct^.

### 2.6 Evaluation of the apoptosis induction and viability of cancer cells upon co-culture with selected LAB strains

#### 2.6.1 Cancer cell culture

A431 human epidermoid carcinoma (Cat. no #21555) and MDA-MB-231 human breast carcinoma (Cat. no #30026) cells were purchased from the Korean Cell Line Bank (Seoul, Republic of Korea). All cells were maintained in Dulbecco’s modified Eagle’s medium (Cat. no #LM001-05; Welgene, Gyeongsan, Republic of Korea) supplemented with 10% fetal bovine serum (Cat. no #S001-01; Welgene, Gyeongsan, Republic of Korea) and 1% P/S (Cat. no #PS-B; Capricorn Scientific, Ebsdorfergrund, Germany). The cells were maintained at 37 °C in a humidified cell incubator with 5% CO_2._

#### 2.6.2 Measurement of cell viability

For cell viability measurements, 1 × 10^3^ cells were seeded into each well of a 96-well culture plate and cultured at the indicated conditions for 72 h. The cells were then analyzed using a Quanti-Max WST-8 cell viability assay kit (Cat. no #QM2500; BIOMAX, Guri, Republic of Korea), according to the manufacturer’s instructions.

#### 2.6.3 Clonogenic assay

Colony formation was confirmed by performing a clonogenic assay. Briefly, A431 and MDA-MB-231 cells were seeded into 96-well culture plates at a density of 1 × 10^3^ cells/well and cultured with the indicated conditions for 96 h. The cells were then fixed with 10% formaldehyde for 20 min. After fixation, the cells were stained with 0.05% crystal violet (Cat. no #C3886, Sigma–Aldrich, MO, USA) for 1 h. The staining area was then measured using ImageJ software (National Institutes of Health, Bethesda, MD, USA).

#### 2.6.4 Immunoblotting analysis

Immunoblotting analysis was performed, as previously described (38). Briefly, cells were harvested and lysed using a cell lysis buffer (50 mM Tris–HCl [pH 7.5], 150 mM NaCl, 1 mM DTT, 0.5 mM EDTA, 0.1% SDS, 1% Triton X-100, 100 μM NaF, 100 μM Na_3_VO_4_, 100 μM Na_4_P_2_O_7_, and cOmplete™, Mini, EDTA-free Protease Inhibitor Cocktail [Cat. no #11836170001, Roche, Basel, Switzerland]) for 30 min at 4 °C. The cell extracts were then centrifuged at 15,000 rpm (4 °C for 15 min), and protein concentrations of the cell lysates were determined using a DC protein assay kit (Cat. no #5000112; Bio-Rad, Hercules, CA, USA). Equal amounts of lysates were resolved using SDS-PAGE, and the separated proteins were transferred onto a nitrocellulose membrane (GE Healthcare Life Sciences, MA, USA). The membrane was blocked with 5% skim milk (BD Difco, NJ, USA) in TBST at room temperature (RT) for 30 min and then incubated with anti-cyclin D1 (Cat. no #sc-8396, Santa Cruz, Dallas, TX, USA), anti c-Myc (Cat. no #3198, Cell Signaling, Danvers, MA, USA), anti-GAPDH (Cat. no #sc-47724, Santa Cruz Biotechnology, Dallas, TX, USA), or anti-PARP (Cat. no #9542, Cell Signaling Technology, Danvers, MA, USA) antibodies at 4 °C overnight. The blots were then incubated with the corresponding horseradish peroxidase-conjugated secondary antibodies (anti-rabbit [Cat. no #RSA1221; BioActs, Incheon, Republic of Korea] or anti-mouse [#RSA1122; BioActs, Incheon, Republic of Korea]) at RT for 2 h. Band intensity was quantified using the ImageJ 1.53e software (National Institutes of Health, MD, USA). Each experiment was repeated at least three times.

### 2.7 Statistical analysis

To determine the statistical differences between the treatment groups and the relevant controls, GraphPad Prism 5 (GraphPad Software, Inc., La Jolla, CA, USA) was used for one-way analysis followed by a Tukey’s *post-hoc* test (39). The experimental results, except for the stress condition test (n = 2), were presented as mean ± standard deviation of three independent replicates (n = 3). For experiments using cells, comparisons between two groups were performed using a two-sided, two-sample Student’s *t*-test. For simultaneous comparisons among more than two groups, one-way ANOVA followed by Tukey’s *post-hoc* test was applied. Statistical analyses were conducted using the SPSS statistical software package (version 12.0; SPSS Inc., Chicago, IL, USA). Differences were considered statistically significant at *p* < 0.05.

## 3 Results

### 3.1 LAB species from ginseng sprouts include Lacticaseibacillus rhamnosus, *Leuconostoc lactis*, and *Leuconostoc mesenteroides*

A total of 688 bacterial isolates were obtained from ginseng sprouts, 364 from the peel (surface), and 324 from the pulp (inner tissues). The physiological characteristics of the 688 isolates (as determined using the BCP test, catalase test, and Gram staining) are presented in Table 1. In total, 17 isolates exhibiting Gram-positive, rod-shaped morphology, BCP-positive, and catalase-negative characteristics were selected as potential LAB strains.

**Table 1:** Characteristics of the 688 bacterial isolates obtained from ginseng sprouts.

Origin	Bacterial isolates	BCP (+) and catalase (−)		Selected strains
		Gram (+)	Gram (−)	
Peel	364	8	16	0
Pulp	324	9	8	5
Total	688	17	24	5

**Figure 1:**
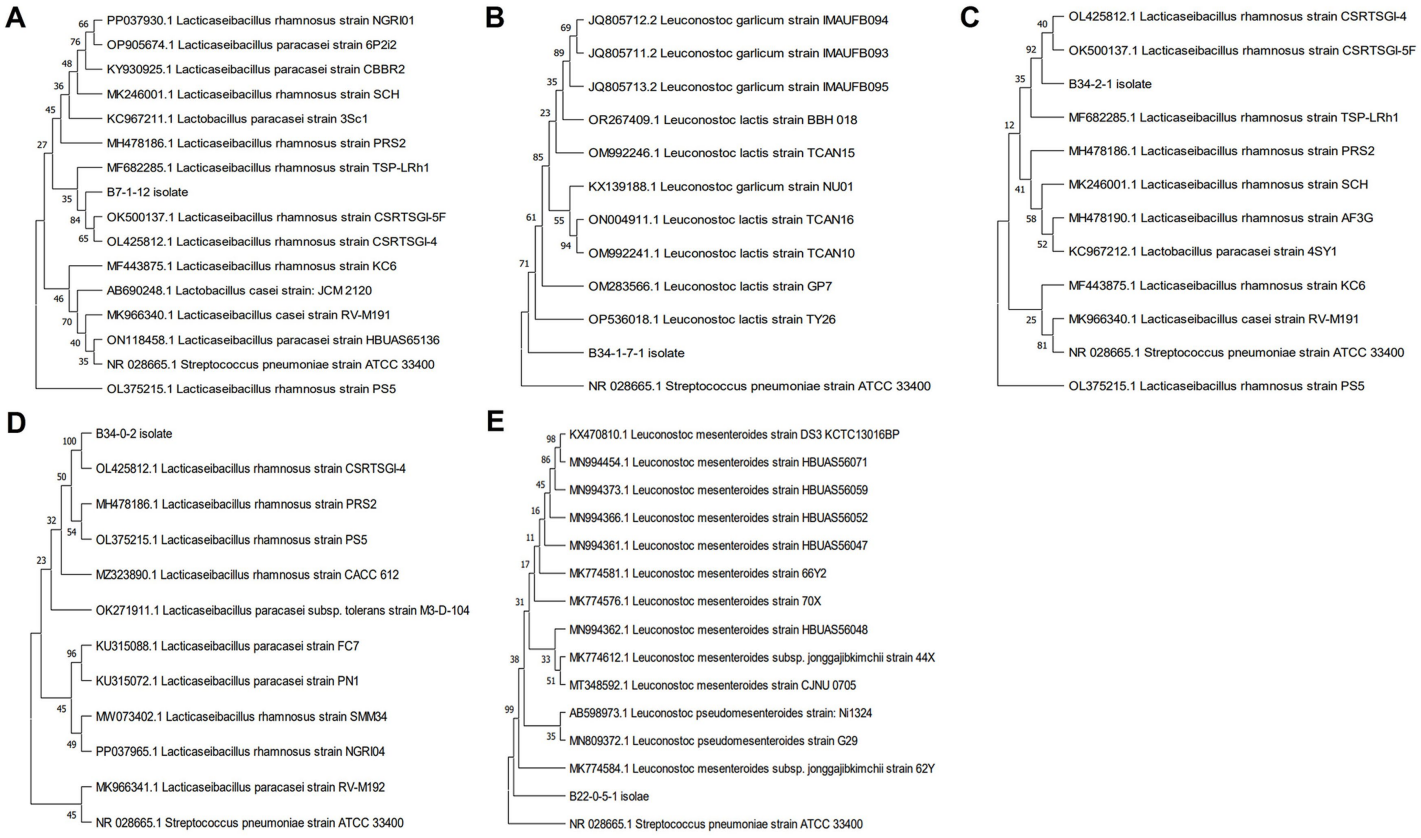
Phylogenetic trees of various distantly related species of bacteria, based on the 16S rRNA gene sequences. Phylogenetic trees were generated using the MEGA 11 software and constructed using the neighbor-joining method. Three bacterial isolates [accession number PQ276990, strain #1 **(A)**; PQ276994, strain #3 **(C)**; and PQ276993, strain #4 **(D)**] exhibited a close phylogenetic relationship with *Lacticaseibacillus rhamnosus*. The bacterial isolates, accession number PQ276991, strain #2 **(B)**, and PQ276995, strain #5 **(E)**, exhibit close phylogenetic relationships with *Leuconostoc lactis* and *L. mesenteroides,* respectively.

To determine if these 17 isolated strains were indeed LAB, we analyzed the isolates through 16S rRNA sequencing. Among the 17 strains, 5 were identified as LAB, representing the species *L. rhamnosus*, *L. lactis* and *L. mesenteroides* (Table 2). The molecular identification of these selected bacterial isolates was further authenticated through phylogenetic analysis (Figure 1). The strains B7112, B3421, and B3402 showed consensus with *L. rhamnosus* strain CSRTSGI-5F (84% bootstrap), *L. rhamnosus* strain CSRTSGI-5F (92% bootstrap), and *L. rhamnosus* strain CSRTSGI-4 (100% bootstrap), respectively. The B34171 isolate showed 71% bootstrap consensus with *L. lactis* strain TY26, and the B22051 isolate showed 99% bootstrap consensus with *L. mesenteroides* subsp. Jonggajibkimchii strain 62Y.

**Table 2:** Accession numbers of the five LAB strains using BLAST (16S rRNA gene).

Strain No.	Strain code	Accession No.	LAB strains	Variety	LED treatment
1	B7112	PQ276990	*Lacticaseibacillus rhamnosus* B7112	V3^*^	Blue light
2	B34171	PQ276991	*Leuconostoc lactis* B34171	V4^*^	White light
3	B3421	PQ276994	*Lacticaseibacillus rhamnosus* B3421	V4	White light
4	B3402	PQ276993	*Lacticaseibacillus rhamnosus* B3402	V4	White light
5	B22051	PQ276995	*Leuconostoc mesenteroides* B22051	V4	UV light

* V3 and V4 indicate the Seosan cultivar of Korean ginseng and landrace ginseng, respectively.

### 3.2 *L. rhamnosus* B7112 (strain #1) and B3402 (strain #4) demonstrates similar resistance to acid and H₂O₂, and greater tolerance to ethanol than LGG

**Figure 2:**
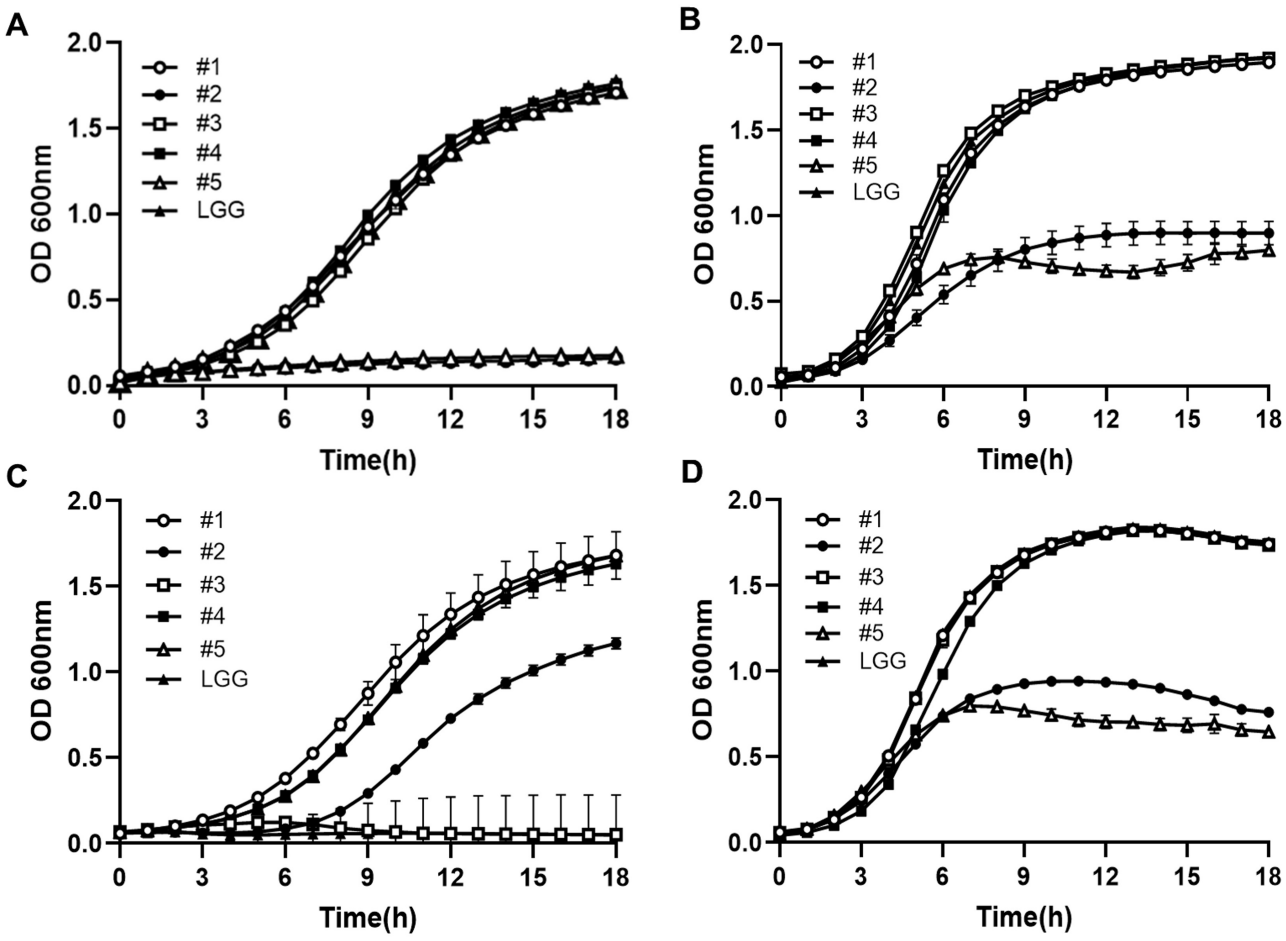
The presence of various stressors (pH, ethanol, and H_2_O_2_) did not affect the growth rate of the five probiotic lactic acid bacteria (LAB) strains. The optical density at 600 nm (OD 600) was monitored for 18 h for the five LAB strains grown in modified MRS broth at 37 °C containing various stressor conditions, such as pH 5 **(A)**, pH 6.5 (**B,** standard MRS as a control), 8% ethanol **(C)**, and 1 mM H_2_O_2_ **(D)**. Strain #1, *Lacticaseibacillus rhamnosus* B7112; #2, *Leuconostoc lactis* B34171; #3, *L. rhamnosus* B3421; #4, *L. rhamnosus* B3402; #5 for *L. mesenteroides* B22051. *L. rhamnosus* GG KCTC 5033 (LGG) was used as a control. The data represent the mean ± standard deviation from two independent replicates (n = 2).

Our results indicated that the five LAB strains exhibited different growth kinetics in response to various pH, H_2_O_2_, and ethanol concentrations when incubated at 37 °C for 18 h (Figure 2). At pH 5, the growth kinetics of the LAB strains improved (Figure 2A). Specifically, strain #4 (B3402) showed the best growth kinetics, while strain #2 (B34171) and strain #5 (B22051) struggled to grow under acidic conditions. Strain #1 (B7112) and strain #3 (B3421) showed moderate growth compared to the control. At pH 6.5, bacterial strains #1, #3, #4, and LGG grew relatively more robustly, while bacterial strains #2 and #5 demonstrated relatively poorer growth performance (Figure 2B). Their growth appeared to plateau at 6 h compared to the other strains with better growth. Under 8% ethanol conditions, bacterial strain #1 grew most efficiently, while strain #3 exhibited minimal growth (Figure 2C); the growth kinetics of the other bacterial strains fell between these two extremes. Under 1 mM H_2_O_2_ conditions, strains #1, #3, and #4 grew better than the other strains (Figure 2D). However, unlike at 1 mM H_2_O_2_, the bacterial strains, including the control (LGG), did not grow appreciably at 10 mM H_2_O_2_ over the 18 h incubation period, suggesting that such conditions are inhibitory to their growth (Figure 2). The growth performance of the five LAB strains and the control LGG strain was sensitive to both pH 4 and 10 mM H_2_O_2_ conditions (data not shown).

### 3.3 The survival of the five selected LAB strains at pH 3 and 0.3% bile salt conditions indicates their potential for gastrointestinal tolerance

**Figure 3:**
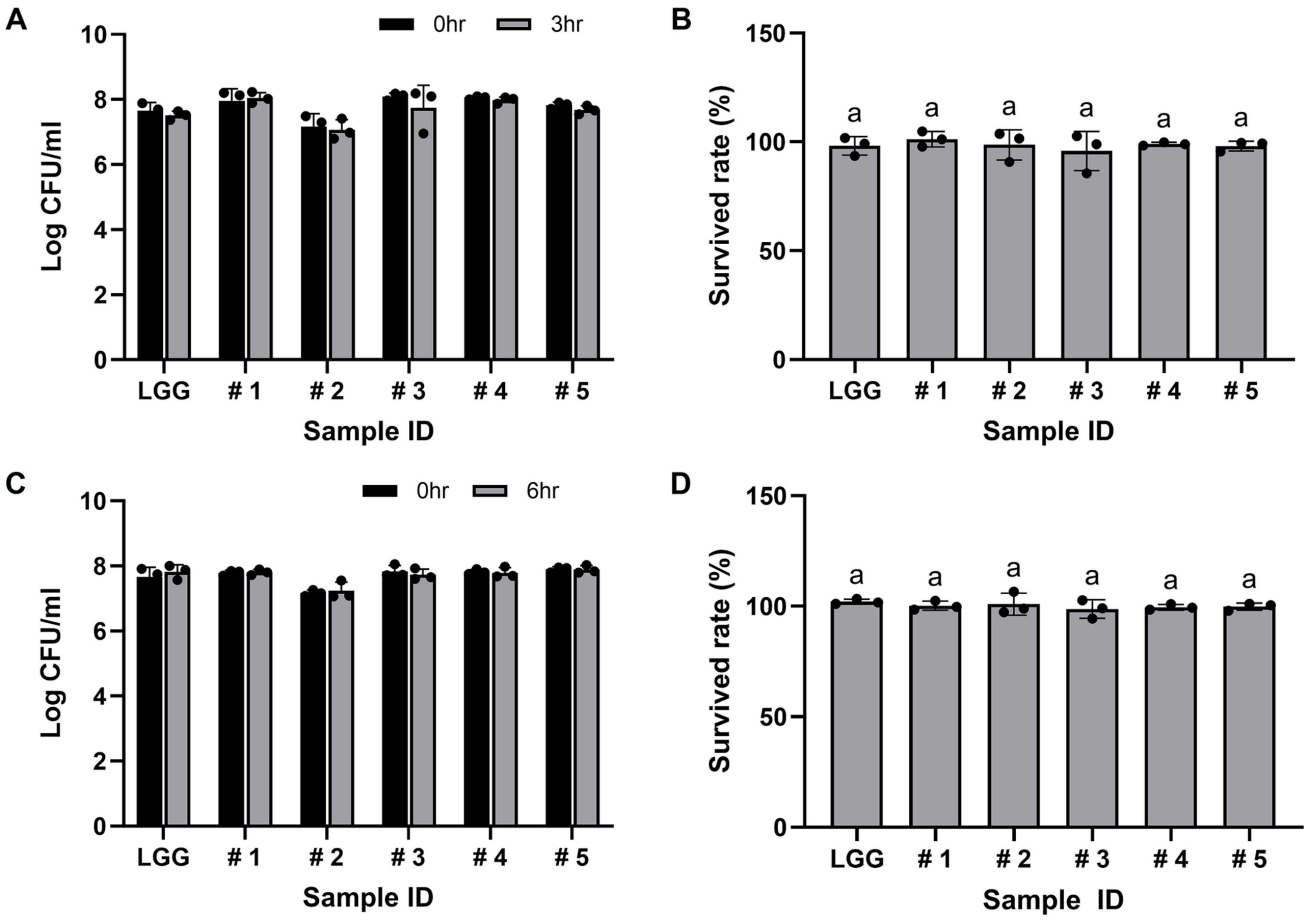
The five lactic acid bacterial (LAB) strains exhibit tolerance to acid and bile salts. **(A)** Viability (log_10_CFU/ml) of the LAB strains at 0 h and 3 h when subjected to pH 3. **(B)** Survival rate (%) of the LAB strains subjected to the pH 3 condition. **(C)** Viability (log_10_ CFU/ml) of the LAB strains when treated with 0.3% bile salt for 0 h and 6 h. **(D)** Survival rate (%) of the strains when treated with 0.3% bile salt. Strain #1, *Lacticaseibacillus rhamnosus* B7112; #2, *Leuconostoc lactis* B34171; #3, *L. rhamnosus* B3421; #4, *L. rhamnosus* B3402; #5 for *L. mesenteroides* B22051. *L. rhamnosus* GG KCTC 5033 (LGG) was used as a positive control. The data represent the mean ± SD from three independent replicates (n = 3). No significant differences in survival rate (%) were observed between LGG and the samples, as determined using the one-way ANOVA followed by the Tukey’s test.

After exposure to low pH (pH 3) conditions, the selected bacterial isolates showed no significant differences in growth, with all exhibiting viabilities of nearly 8 log_10_ CFU/ml and survival rates above 95% (Figures 3A,B). Among these strains, *L. rhamnosus* B7112 (#1) showed the highest resistance to low pH conditions, whereas *L. rhamnosus* B3421 (#3) exhibited the lowest resistance (Figure 3B). Compared with the control (LGG), no significant differences were observed among all the strains. Furthermore, the five LABs tested for their tolerance to bile salts showed no log reduction or difference in survival rate (Figures 3C,D), and had a viability rate comparable to the control (LGG) in the bile tolerance assay. Overall, our results indicate that the selected bacterial isolates are resistant to harsh gastrointestinal conditions, highlighting their potential as safe and functional probiotic candidates.

### 3.4 Confirmation of the safety of the five LAB isolates through antibiotic sensitivity and non-hemolysis assays

All five strains, as well as the LGG control, were susceptible to six tested antibiotics, EM, TC, CM, GM, BP, and CF (Table 3). Any strain with a minimum inhibitory concentration (MIC) greater than the breakpoint was considered resistant to the tested antimicrobial. All the bacterial strains showed values lower than the European Food Safety Authority (EFSA) cut-off standard values (40) except strain #5, which showed values equal to the EFSA values against antibiotic CF. We observed that the five LAB strains were more sensitive to CM, GM, and BP, but comparatively more resistant to TC than LGG (Table 3). Moreover, a clear zone around the corresponding colonies on blood agar indicates β-hemolysis, while no zones around colonies indicate γ-hemolysis. The hemolytic activity test results showed that all five isolates exhibited γ-hemolysis, as no visible change around the colonies was observed (Supplementary Figure S1).

**Table 3:** Representative responses of the five lactic acid bacterial isolates against six different antibiotics on MRS agar plates.

Antibiotics	Breakpoint (μg/ml)^*^	Sensitivity to the antibiotics^*^/MIC (μg/ml)					
		LGG	#1	#2	#3	#4	#5
Erythromycin (EM)	1	S/0.13	S/0.016	S/0.75	S/0.125	S/0.023	S/0.125
Tetracycline (TC)	8	S/0.19	S/2.0	S/0.38	S/1.5	S/0.38	S/0
Clindamycin (CM)	1	S/1.0	S/0.094	S/0.016	S/0.25	S/0.19	S/0.125
Gentamicin (GM)	16	S/8.0	S/0.16	S/0.16	S/2.0	S/8.01	S/2.0
Benzylpenicillin (BP)	1	S/0.38	S/0.25	S/0.094	S/0.25	S/0.19	S/0
Ciprofloxacin (CF)	4	S/3.0	S/0.5	S/0.5	S/3.0	S/1.0	S/4.0

* The breakpoint was adapted from the European Food Safety Authority (EFSA).

A strain is susceptible (S) when its growth is inhibited at a concentration equal to or lower than the established cut-off value for a specific antimicrobial (S ≤ x mg/L) and resistant (R) when it is able to grow at a concentration higher than the established cut-off value for a specific antimicrobial (R > x mg/L) (40). Strain #1, *Lacticaseibacillus rhamnosus* B7112; #2, *Leuconostoc lactis* B34171; #3, *L. rhamnosus* B3421; #4, *L. rhamnosus* B3402; #5, *L. mesenteroides* B22051. *L. rhamnosus* GG KCTC 5033 (LGG) was used as a control.

These results indicate that the selected LAB strains were susceptible to all the tested antibiotics (EM, TC, CM, GM, BP, and CF) and showed no hemolytic activity. Therefore, these strains can be considered safe and suitable probiotic candidates derived from ginseng sprouts.

### 3.5 The five probiotic candidates displayed inhibitory activity against the pathogenic bacteria, *E. coli* and *S. aureus*

The inhibitory activity of the five candidate probiotic strains was evidenced by the size of zones of inhibition against common intestinal pathogens (Table 4). All isolates exhibited antimicrobial activity against two known pathogens, *E. coli* and *S. aureus*. Bacterial isolates #1, #3, and #4 produced particularly wider inhibition zones against *S. aureus* than LGG and lactic acid, although the difference was not statistically significant. Moreover, the antibacterial activity of the five LAB strains was similar to the positive controls in the case of *E. coli*. Among these LAB strains, isolate # 3 exhibited the strongest antibacterial activity, although the difference was not statistically significant.

**Table 4:** Antibacterial activity of selected lactic acid bacteria (LAB) strains against *Escherichia coli* and *Staphylococcus aureus*.

Strains	Antimicrobial activity (mm)	
	*Escherichia coli*	*Staphylococcus aureus*
LGG	15.36 ± 2.92^a^	11.53 ± 2.00^ab^
P/C	14.85 ± 3.01^a^	10.33 ± 1.46^abc^
N/C	8.00 ± 0.00^b^	8.00 ± 0.00^d^
#1	12.87 ± 2.64^a^	12.93 ± 1.23^a^
#2	13.10 ± 1.27^a^	8.34 ± 0.59^cd^
#3	13.16 ± 0.59^a^	12.66 ± 1.58^ab^
#4	12.76 ± 0.62^a^	12.66 ± 1.41^ab^
#5	13.57 ± 0.41^a^	9.06 ± 1.83^bd^

LGG (KCTC 5033) and lactic acid in MRS broth were used as positive controls (P/C), and MRS broth was used as a negative control (N/C). The data represent the mean ± standard deviation from three independent replicates (n = 3). Different superscript letters represent significant differences (*p* < 0.05) as calculated using the one-way ANOVA followed by Tukey’s test.

### 3.6 The five LAB isolates, especially *L. lactis* B34171 exhibits auto-aggregation ability and hydrophobicity suggestive of probiotic potential

**Figure 4:**
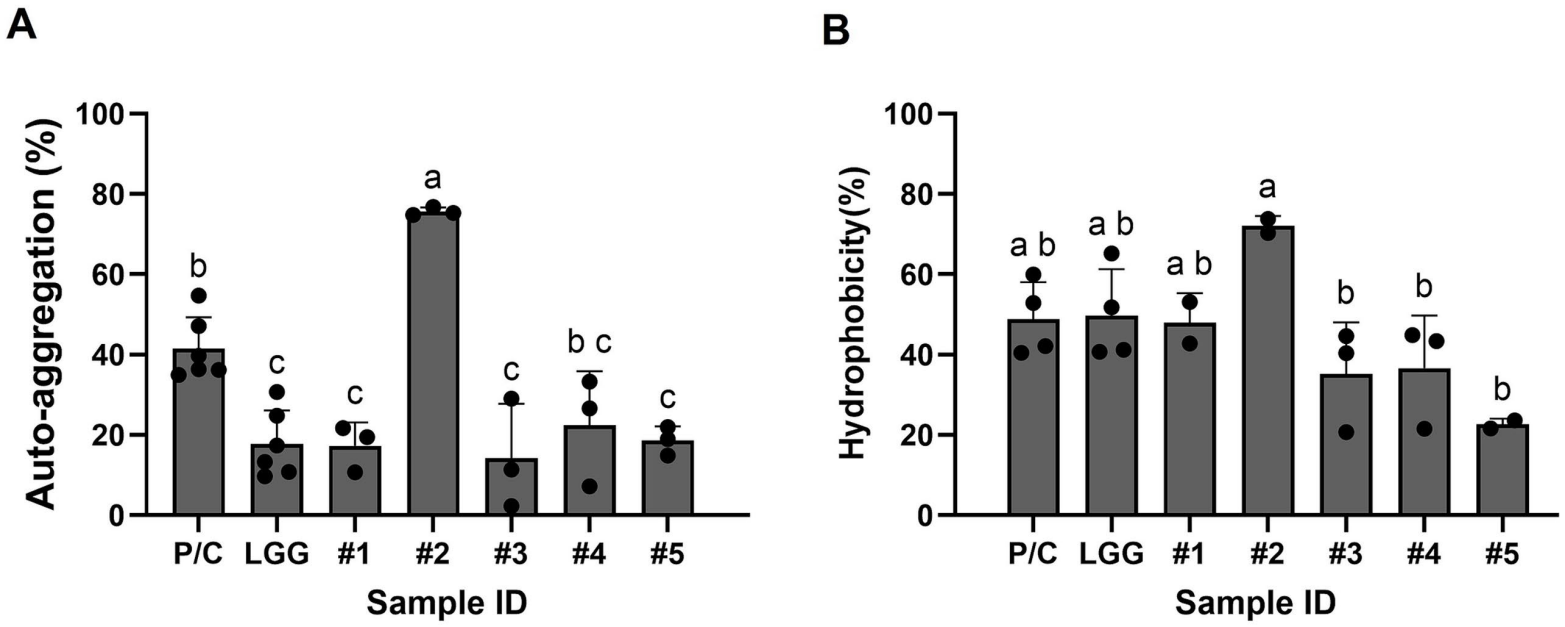
Certain candidate probiotic strains showed higher adhesion ability compared to LGG. Shown are the % auto-aggregation **(A)** and % hydrophobicity **(B)** values of the strains, which were calculated as [1 − (Initial OD_600 /_ Final OD_600_)] × 100. Strain #1, *Lacticaseibacillus rhamnosus* B7112; #2, *Leuconostoc lactis* B34171; #3, *L. rhamnosus* B3421; #4, *L. rhamnosus* B3402; #5, *L. mesenteroides* B22051. *L. rhamnosus* GG KCTC 5033 (LGG) and *L. acidophilus* KCTC 3164 were used as positive controls. The data represent the mean ± standard deviation from three independent replicates (n = 3). Different superscript letters represent significant differences (*p* < 0.05) as calculated using the one-way ANOVA followed by Tukey’s test.

In this study, five candidate probiotic strains exhibited aggregation ability ranging from 14.2 to 75.6% after approximately 5 h of incubation at 37 °C (Figure 4). Additionally, the adhesive and affinity potential of these strains, as evaluated through cell surface hydrophobicity using ethyl acetate, exhibited high affinity rates, ranging between 22.6 and 72.0%. In particular, strain #2 (B34171) showed the highest auto-aggregation ability and hydrophobicity among all tested strains, including the positive control and LGG, and was statistically significant. Compared with the LGG strain, the four LAB strains except strain #2 (B34171) showed similar auto-aggregation ability but lower hydrophobicity percentages. These results reveal that our five candidate probiotic strains have the potential to adhere to intestinal epithelial cells, allowing them to colonize the gastrointestinal tract ecosystem.

### 3.7 DPPH radical scavenging activity demonstrates the antioxidant potential of the five probiotic LAB strains

**Figure 5:**
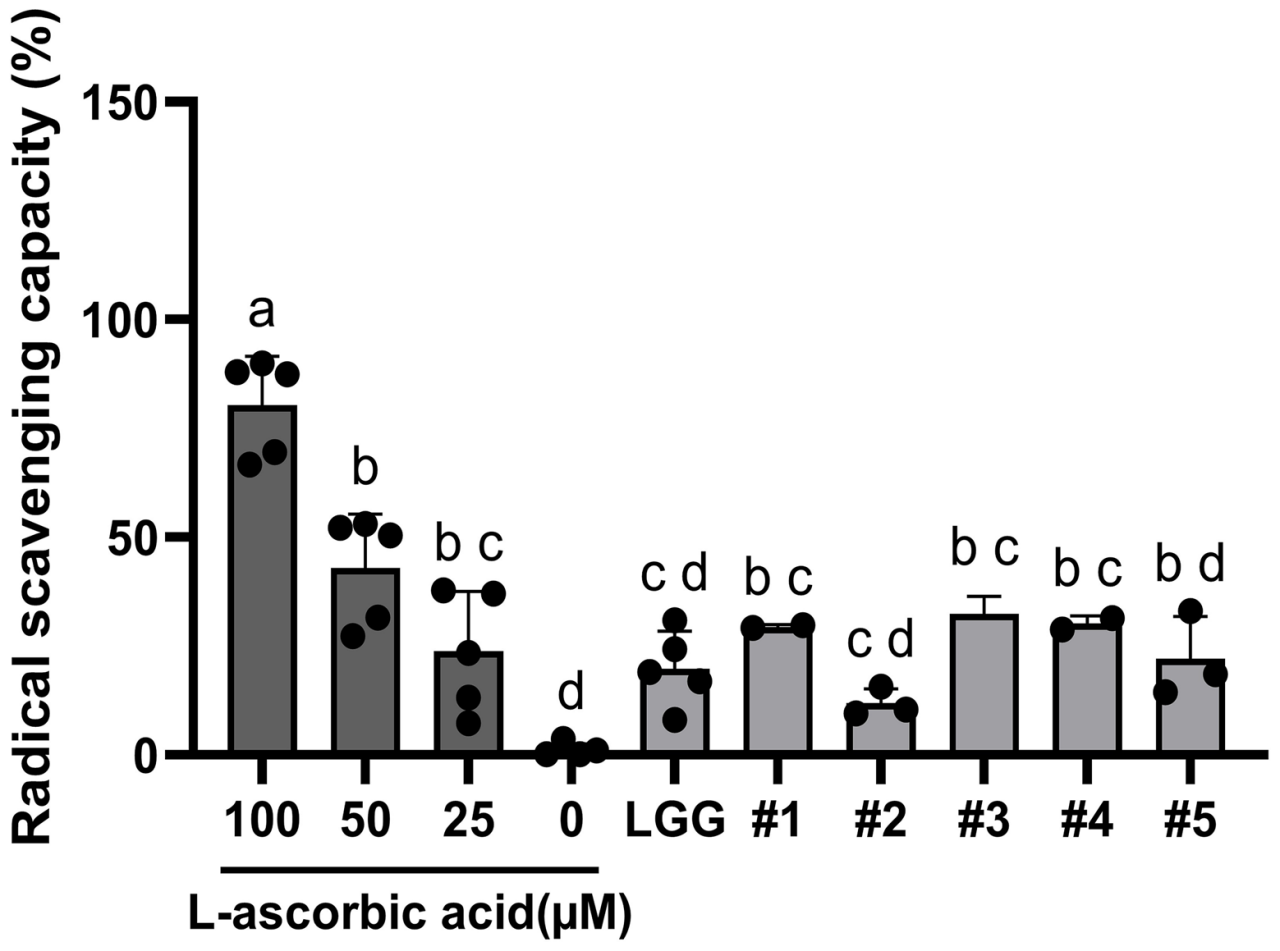
The five bacterial isolates from ginseng sprouts showed antioxidant properties as evidenced by their 1,1-diphenyl-2-picrylhydrazyl (DPPH) free radical scavenging activity. Strain #1, *Lacticaseibacillus rhamnosus* B7112; #2, *Leuconostoc lactis* B34171; #3, *L. rhamnosus* B3421; #4, *L. rhamnosus* B3402; #5, *L. mesenteroides* B22051. *L. rhamnosus* GG KCTC 5033 (LGG) and L-ascorbic acid were used as positive control. The data represent the mean ± standard deviation from three independent replicates (n = 3). Different superscript letters represent significant differences (*p* < 0.05) as calculated using the one-way ANOVA followed by Tukey’s test.

In this study, all tested strains exhibited DPPH scavenging activity (Figure 5). All candidate strains exhibited higher or the same scavenging activity of DPPH free radicals compared to that of LGG. Of these, strain #3 (B3421) demonstrated the highest DPPH scavenging activity among the strains, whereas strain #2 (B34171) exhibited lower activity than the other strains. The DPPH scavenging activity of the five strains was significantly lower than that of 100 μM L-ascorbic acid, but not significantly different from that of 25 μM L-ascorbic acid used as a control, particularly for three strains of *L. rhamnosus*. These results suggest that the five LAB strains modulate inflammation by reducing ROS-induced damage to DNA, proteins, lipids, and small molecules (41).

### 3.8 Protein extracts (PE) of *L. rhamnosus* B3421 (strain #3) and B3402 (strain #4) exhibited strong reducing effects against TNF-α expression in RAW264.7 cell

**Figure 6:**
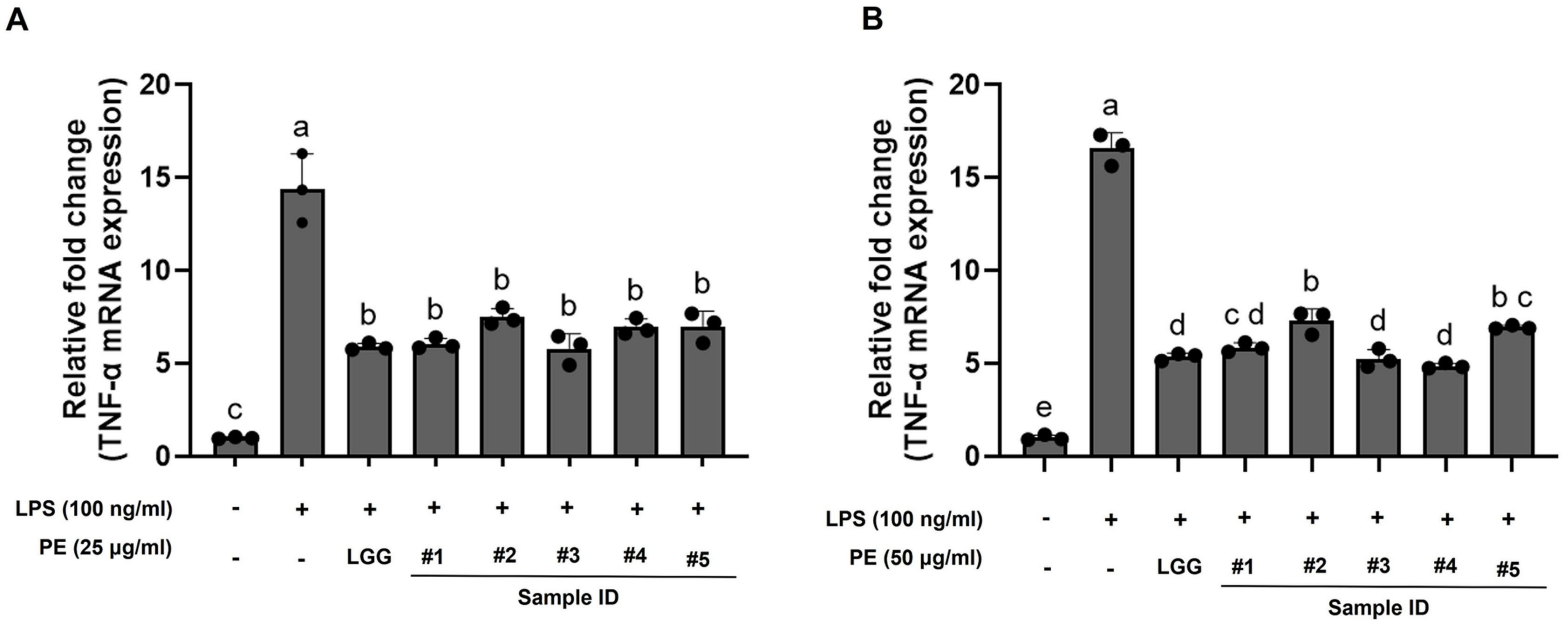
The relative mRNA expression of the pro-inflammatory cytokine TNF-α was inhibited by treatment with protein extracts (PE) from the candidate lactic acid bacterial strains, as assessed using RT-qPCR. Relative mRNA expression of TNF-α (fold change) upon treatment with 25 μg/ml **(A)** and 50 μg/ml **(B)** of PE from the five probiotic candidates. Strain #1, *Lacticaseibacillus rhamnosus* B7112; #2, *Leuconostoc lactis* B34171; #3, *L. rhamnosus* B3421; #4, *L. rhamnosus* B3402; #5, *L. mesenteroides* B22051. *L. rhamnosus* GG KCTC 5033 (LGG) was used as a positive control. The data represent the mean ± standard deviation from three independent replicates (n = 3). Different superscript letters represent significant differences (*p* < 0.05) as calculated using the one-way ANOVA followed by Tukey’s test.

The effect of treatment with LAB on LPS-stimulated RAW264.7 was evaluated for TNF-α mRNA expression, suggesting an inflammatory response. Notably, treatment of RAW264.7 cells with PE from the selected LAB strains resulted in the suppression of TNF-α expression compared to the LPS-stimulated group (Figure 6). In the 25 μg/ml PE treatment group, selected LAB strains inhibited TNF-α mRNA expression in LPS-stimulated RAW264.7 cells, similar to that of LGG. TNF-α mRNA expression was not significantly reduced with a further increase in LAB-PE treatment concentration. TNF-α mRNA levels in RAW264.7 cells treated with 50 μg/ml PE from strain #3 (B3421) and #4 (B3402) were reduced compared to those in the group treated with 25 μg/ml PE. In the 50 μg/ml PE treatment, TNF-α mRNA levels after treatment with the PE of strains #2 (B34171) and #5 (B22051) were higher than in the LGG and other LAB strains. These findings indicate that *L. rhamnosus* B3421 (strain #3) and B3402 (strain #4) exhibited strong reducing effects against LPS-induced inflammatory responses in RAW264.7 cells, similar to that induced by LGG.

### 3.9 PE from the five probiotic LAB strains inhibited cancer cell proliferation and induced apoptosis in human epidermoid and breast carcinoma cells

**Figure 7:**
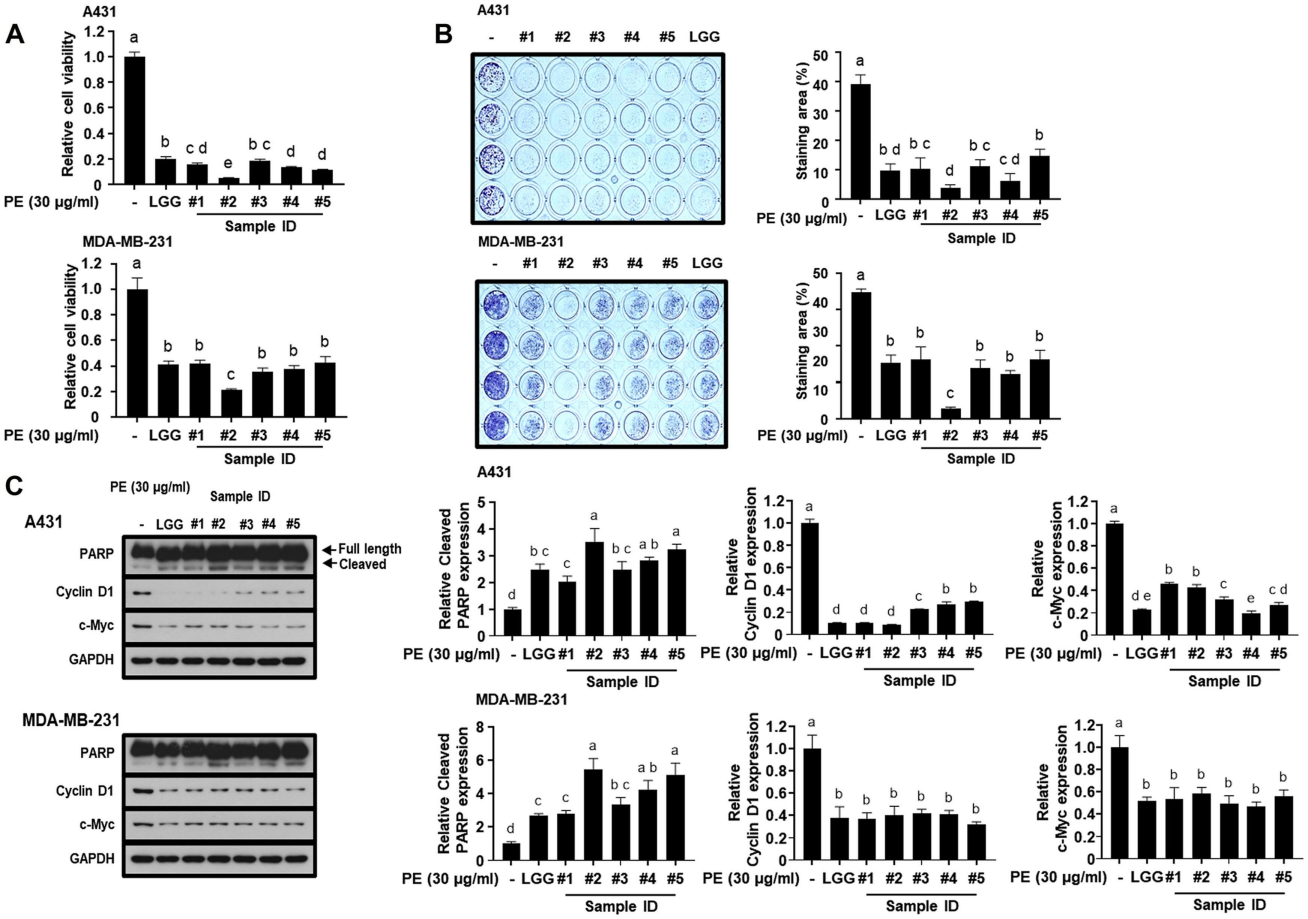
Treatment with protein extracts (PE) of the five candidate probiotic strains affects the viability and proliferation of cancer cells. A431 and MDA-MB-231 cells were cultured with or without the indicated lactic acid bacteria (LAB) PE for 72 h. Cell viability was measured using the WST-8 assay **(A)**. A431 and MDA-MB-231 cells were cultured with or without the indicated LAB PE for 96 h **(B)**. The cells were fixed with formalin and then stained with crystal violet. Representative images of the plates (left panel) and quantification graphs (right panel) are shown. A431 and MDA-MB-231 cells were cultured with or without the indicated LAB PE for 48 h **(C)**. The cells were harvested, the proteins were extracted, and subjected to immunoblotting analyses with the indicated antibodies. Representative image of the blots (left panel) and quantification graph (right panel) are shown. Strain #1, *Lacticaseibacillus rhamnosus* B7112; #2, *Leuconostoc lactis* B34171; #3, *L. rhamnosus* B3421; #4, *L. rhamnosus* B3402; #5, *L. mesenteroides* B22051. *L. rhamnosus* GG KCTC 5033 (LGG) was used as a positive control. Data are presented as mean ± standard deviation of three independent experiments (n = 3). Different superscript letters represent significant differences (*p* < 0.05) as calculated using the one-way ANOVA followed by Tukey’s test.

To determine the effects of the selected LAB strains on cancer cell viability, we treated A431 human epidermoid carcinoma cells and MDA-MB-231 human breast carcinoma cells with PE from the strains and subjected them to the WST-8 assay. As shown in Figure 7A, the LAB strain significantly inhibited the viability of all tested cancer cells, which were comparable to or better than those of the control (LGG). In addition, the results of the clonogenic assay showed that treatment with LAB PE markedly decreased the number of colonies (Figure 7B). Consistent with the cell viability and colony formation data, immunoblotting analysis revealed that the expression levels of the cell proliferation markers, cyclin D1 and c-Myc, were significantly downregulated while that of the apoptotic marker cleaved PARP was significantly upregulated upon treatment with the LAB PE (Figure 7C). These results indicate that the LAB PE exerts anti-cancer effects by inhibiting the proliferation of cancer cells and inducing their apoptosis.

## 4 Discussion

The identification of novel probiotic strains from plant sources is important in the field of functional foods, as adaptation to bioactive compound-rich environments may generate unique metabolic traits not found in dairy- or human-derived probiotic strains. *Panax ginseng*, widely used in East Asia for medicinal and dietary purposes, is now recognized as a valuable niche that supports a unique plant-associated microbial ecosystem (19). Ginseng sprouts are recognized as a potentially valuable food source, as they provide a rich source of bioactive compounds with antioxidant and anti-cancer properties, which supports the idea that probiotics with such characteristics could be derived from ginseng sprouts (19). Recently, LAB strains associated with medicinal plants, such as those from the rhizosphere of the medicinal plants *Ocimum tenuiflorum*, *Azadirachta indica*, and *Ficus carica*, have been extensively isolated, and some strains were characterized as potential probiotics exhibiting antioxidant, anti-inflammatory, and anti-diabetic effects (42). In the current study, five LAB strains were identified from ginseng sprouts and systematically characterized for their probiotic properties, anti-inflammatory efficacy, and anti-cancer potential, supporting this knowledge.

Consistent with previous studies (19, 20), we successfully isolated LAB strains from ginseng sprouts. Notably, two strains of *Leuconostoc* spp. were isolated, and they were distinct from previously reported ginseng-derived probiotic species (20–23). These results suggest that ginseng sprouts cultivated in smart-farm hydroponic systems provide a promising reservoir for uncovering unreported probiotic candidates with novel functional potential. This controlled growth environment system minimizes external contamination and enables precise characterization of phyllosphere-associated microbiota, thereby facilitating the discovery of unique plant-adapted LAB strains.

Subsequent 16S rRNA sequencing followed by phylogenetic analysis identified *L. rhamnosus* (strains #1, #3, and #4), *L. lactis* (strain #2), and *L. mesenteroides* (strain #5). Both *Lacticaseibacillus* and *Leuconostoc* spp. are known as the most common species of probiotics (3, 4). For a long time, strains of *L. rhamnosus* have been used as probiotics for infants and children in a wide range of different probiotic products, which are marketed in many countries. One of the most studied strains is *L. rhamnosus* GG, which is well-tolerated and safe for infants and children (43–45). Moreover, *L. mesenteroides* is one of the most well-studied probiotics. Its characteristics, such as antioxidant activities and immunity-improving properties, have been reported in several studies (46, 47). However, because each strain is different, it is essential to select and identify probiotics with the optimal characteristics for the desired applications.

Despite the difficulties encountered in reliably characterizing probiotic strains using *in vitro* methods, the initial screening of strains in this manner remains a useful preliminary step in the detection of probiotic candidates. The selected bacterial strains isolated from ginseng sprouts, three *L. rhamnosus* strains, and two *Leuconostoc* strains, showed good probiotic characteristics. The growth pattern of these five LAB strains under various stress conditions was evaluated; they were able to maintain viability at pH 5 or 6.5, 8% ethanol (except for strain #3), and 1 mM H_2_O_2_ better than the positive and negative controls employed in our investigation. The survival rate of the five presumptive probiotics under conditions simulating the gastrointestinal tract was identified through tolerance tests to bile salts and acidic environments. The survival rate of the five isolates was above 95% in both acidic (pH 3) and 0.3% bile salt conditions, which did not differ significantly from that of the control (*L. rhamnosus* GG). These findings are in line with earlier research indicating that probiotic strains exhibit a high survival rate at pH 3 and 0.3% bile salt conditions (48, 49), clearly suggesting that the five presumptive LAB strains can withstand conditions that mimic the gastric intestinal environment of humans.

Another important factor in assessing probiotic potential is antibacterial activity (20). In this study, all tested probiotic strains exhibited measurable antibacterial effects against *E. coli* and *S. aureus*, both of which are intestinal pathogens associated with intestinal disorders (50). The strain-specific variation in inhibition observed here is in agreement with previous studies (51). These findings suggest that the antimicrobial activity may be mediated by bioactive substances generated by LAB, notably organic acids, H_2_O_2_, and bacteriocins (52). These antimicrobial activities, combined with the excellent adhesion capacity, imply that all five strains may function as probiotics beneficial to human health by suppressing pathogen colonization in the gastrointestinal tract (53).

Bacteria for human and animal consumption must first be evaluated for Generally Recognized As Safe (GRAS) and/or Qualified Presumption of Safety (QPS) status (54). According to the EFSA guidelines, antibiotic susceptibility testing is the first step in safety assessment. Consistent with previous studies (33, 55), all five LAB strains were found to be sensitive to EM, TC, CM, GM, BP, and CF. Notably, all potential probiotic LAB strains exhibited equal susceptibility to all six antibiotics tested compared to LGG, which is reported to be safe (56). Moreover, there was an absence of hemolysis on blood agar plates, indicating that all five strains were γ-hemolytic. The blood agar test is widely used to evaluate the ability of bacteria to lyse red blood cells, as hemolytic activity can be associated with pathogenicity. The absence of hemolytic activity observed in this study indicates that the tested lactic acid bacteria strains are non-hemolytic (γ-hemolysis), which is a critical factor in assessing their safety as potential probiotics (57). This is consistent with previous reports demonstrating the non-hemolytic nature and safety of *Lactobacillus fermentum* strain PRI 29 (58), *Lacticaseibacillus* strains KF7 and LGG (59), as well as yeast and *Lactobacillus* isolates from fermented foods in North-eastern India (60). These findings collectively support the safety of the tested strains as potential probiotics.

Other essential criteria for selecting probiotic strains include cell surface hydrophobicity, auto-aggregation, and epithelial cell adherence, which are required for adhesion to target sites of the gastrointestinal tract (61). Probiotics exert their beneficial effects partly by modulating the gut microbiota and by attaching to the epithelial and mucosal layers (62). Therefore, intestinal adhesion is considered a key functional characteristic when evaluating the probiotic potential of bacteria. In the present study, strain #2 showed the highest auto-aggregation ability (75.6%) and hydrophobicity (72%) among all tested strains. Moreover, the auto-aggregation capacity of the four other LAB strains ranged from 14.2 to 22.4%, which was similar to that of *L. rhamnosus* (35). As reported in previous studies (53, 63), the auto-aggregation levels of the commercial probiotic strains *L. rhamnosus* GG, GR-1, and *L. acidophilus* La-5 were found to be 41.4 ± 3.3%, 15.2 ± 0.6%, and 15.9 ± 1.1%, respectively. Furthermore, auto-aggregation abilities ranging from 11.5 to 29.0% have been observed in certain *Lactobacillus* and *Bifidobacterium* strains, as described by Krausova et al. (64). Despite having slightly lower levels of aggregation and hydrophobicity compared to the presumptive probiotic LAB strains in this study (except for strain #2), the results did not significantly differ from those of LGG. The selected LAB strains, especially strain #2, exhibit high self-aggregation ability and hydrophobicity; therefore, they may be sufficient to exert a beneficial effect on the human body as probiotics.

Because of its ease of use, rapidity, sensitivity, and reproducibility, the DPPH radical scavenging assay is widely employed to evaluate antioxidant activity (65). In this study, the five LAB isolates exhibited DPPH scavenging activities ranging from 12 to 32% (Figure 5), which were relatively similar to that of the control (25 μM L-ascorbic acid). *L. rhamnosus* B7112 (strain #1), B3421 (strain #3), and B3402 (strain #4) showed greater DPPH scavenging activities (29.49, 32.41, and 30.15%, respectively) than the reference strain, LGG (19.86%). Our results align with earlier reports (66), indicating that the physiological properties of strains can differ according to their origin, even among isolates of the same species. The pronounced DPPH antioxidant activity of *L. rhamnosus* B3421 may be attributed to the presence of three genes associated with oxidase activity detected on its chromosome (67). This finding agrees with previous reports demonstrating that the DPPH scavenging activity of ginseng-derived *L. reuteri* KGC 1901 was 13% (23), and that 15 strains purified from fermented food (“Jiangshui” and pickles) or feces exhibited 28.81–82.75% DPPH scavenging activity (68). Previous studies also reported that probiotics have the ability to scavenge free radicals through several mechanisms, thereby improving host health following colonization in the human gastrointestinal tract (69, 70). Our findings suggest that the five LAB isolates from ginseng sprouts are potential probiotics capable of preventing diseases related to oxidative stress. These antioxidant effects (71) were reported to be strain-specific, depending on their cell wall composition, enzyme, and metabolite production capabilities.

In addition, our study demonstrated that the five putative probiotics effectively mitigate the LPS-induced expression of TNF-α in RAW 264.7 macrophages. TNF-α, IL-6, and IL-1β are key pro-inflammatory cytokines secreted by macrophages and endothelial cells. They contribute to inflammatory cell aggregation and activation, stimulate the release of inflammatory mediators, stimulate fever production, and exacerbate inflammatory responses (72, 73). Consistent with our results, a previous study demonstrated that *L. plantarum* T1 CFS can negatively regulate pro-inflammatory cytokine expression to improve the inflammatory response caused by LPS (74). Studies have also shown that probiotics can exert anti-inflammatory effects by inhibiting the production of pro-inflammatory cytokines such as TNF-α, IL-10, and IL-12. Moreover, the results of many *in vitro* studies indicate the beneficial properties of probiotics in modulating the proliferation and apoptosis of various types of cancer cells, including gastric, colonic, and myeloid leukemia cells (75). Previous research demonstrated that *L. rhamnosus* markedly suppressed the proliferation of B-CPAP cancer cells (76) and was effective in inhibiting mammary tumor growth in cancer cell-transplanted mice (77). *L. mesenteroides* has also been reported to induce apoptosis in colon cancer cells (78). Consistent with previous findings (76–78), our results indicate that the extracts from the five LAB strains exert anti-cancer effects by inhibiting the proliferation and inducing apoptosis of cancer cells. These findings suggest that the five potential probiotic strains identified in this study may contribute to reducing inflammation and improving human health.

Although the current study demonstrates promising *in vitro* anti-inflammatory and anti-cancer activities of LAB strains isolated from ginseng sprouts, there are still several limitations. First, while pH and osmotic pressure are factors affecting the growth of lactic acid bacteria, their changes during growth were not measured. Future measurements of the physiological activity of isolated lactic acid bacteria should include changes in these factors. Second, mechanistic studies on the antibacterial and antioxidant properties of isolated lactic acid bacteria are needed. For example, examining the metabolites or cell wall components of specific isolated bacteria to determine their effects on antibacterial and antioxidant properties is necessary. Third, strain-specific safety assessments that comply with the regulatory standards of each country will still be required prior to commercialization, although LAB are generally recognized as safe (GRAS) owing to their long history of use in fermented foods. Finally, since the functionality of the isolated LAB was verified only *in vitro*, further *in vivo* studies are required to provide clinical evidence.

## 5 Conclusion

This study highlights ginseng sprouts as a rich source of diverse probiotics, including three strains of *L. rhamnosus* and two *Leuconostoc* strains. In particular, *L. rhamnosus* B3421 (strain #3) and B3402 (strain #4) exhibited notable anti-inflammatory activity, suggesting their strong potential as functional probiotics for improving intestinal health and preventing inflammation-associated disorders. Additionally, as *L. lactis* B34171 (strain #2) exhibited anti-cancer and apoptotic effects on the A431 and MB-231 cell lines, suggesting its promise as a candidate for the treatment and control of breast cancer. Future studies should aim to validate these functional effects in animal models and assess their mechanisms and applicability for human and animal health.

## Data Availability

The 16S rRNA gene sequences of the B7112, B34171, B3421, B3402, and B22051 strains were deposited in the NCBI GenBank database under the accession numbers PQ276990, PQ276991, PQ276994, PQ276993, and PQ276995, respectively.
