# Supplementary figures and images for: Isolation and identification of lactic acid bacteria from ginseng sprouts and research on their probiotic, anti-inflammatory, and anti-cancer activity
Source: Front Nutr. 2026 Jan 5;12:1718524. doi: 10.3389/fnut.2025.1718524 (PMC12812585; doi:10.3389/fnut.2025.1718524)

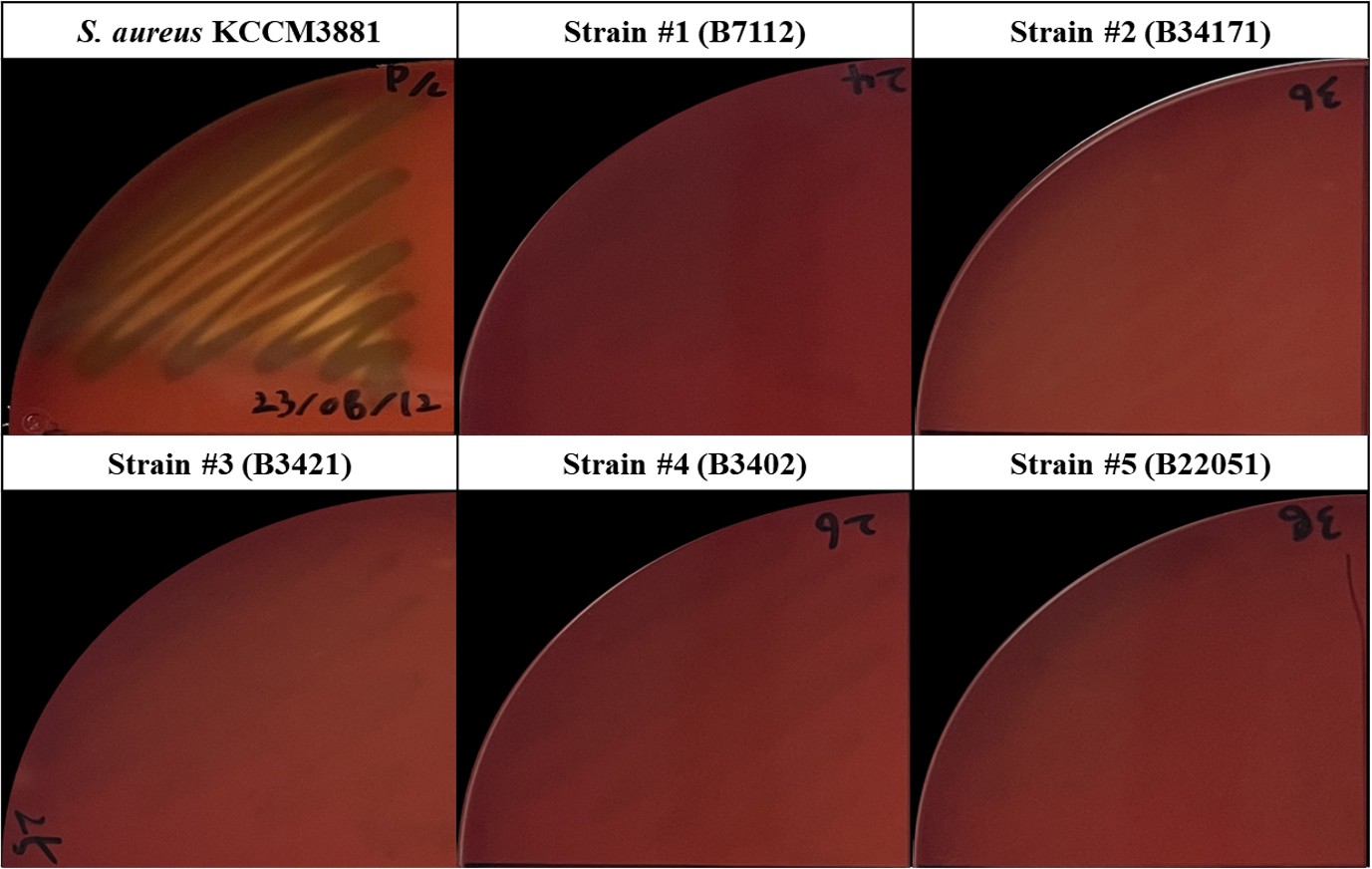

Supplement: Supplementary file 1 [file Image_1.JPEG]
